# An alternative smooth particle hydrodynamics formulation to simulate chemotaxis in porous media

**DOI:** 10.1007/s00285-016-1049-6

**Published:** 2016-08-27

**Authors:** Diego Avesani, Michael Dumbser, Gabriele Chiogna, Alberto Bellin

**Affiliations:** 1 Department of Civil, Environmental and Mechanical Engineering, University of Trento, via Mesiano 77, 38123 Trento, Italy; 2 Chair of Hydrology and River Basin Management, Technical University of Munich, Arcisstr. 21, 80333 München, Germany

**Keywords:** Smooth particle hydrodynamics (SPH), Chemotaxis, Meshfree WENO, Lagrangian particle methods, 76-xx Fluid mechanics, 76M25 Other numerical methods, 76Zxx Biological fluid mechanics, 76Z99 None of the above, but in this section, 92C17 Cell movement (chemotaxis, etc.)

## Abstract

Chemotaxis, the microorganisms autonomous motility along or against the concentration gradients of a chemical species, is an important, yet often neglected factor controlling the transport of bacteria through saturated porous media. For example, chemotactic bacteria could enhance bioremediation by directing their own motion to residual contaminants trapped in low hydraulic conductive zones of contaminated aquifers. The aim of the present work is to develop an accurate numerical scheme to model chemotaxis in saturated porous media and other advective dominating flow systems. We propose to model chemotaxis by using a new class of meshless Lagrangian particle methods we recently developed for applications in fluid mechanics. The method is based on the Smooth Particle Hydrodynamics (SPH) formulation of (Ben Moussa et al., Int Ser Numer Math, 13(1):29–62, 2006), combined with a new Weighted Essentially Non-Oscillatory (WENO) reconstruction technique on moving point clouds in multiple space dimensions. The purpose of this new numerical scheme is to fully exploit the advantages of SPH among traditional mesh-based and mesh-free schemes and to overcome drawbacks related to the use of standard SPH for modeling chemotaxis in porous media. First, we test the new scheme against analytical reference solutions. Then, under the assumption of complete mixing at the Darcy scale, we perform two-dimensional conservative solute transport simulations under steady-state flow conditions, to show the capability of the proposed new scheme to model chemotaxis.

## Introduction

Chemotaxis is the ability of biological cells and organisms to move along or against chemical concentration gradients [see for example Alt 1980; Erban and Othmer 2004; Hilpert 2005; Hillen and Painter 2009; Long and Ford 2009; Pedit et al. 2002], and it can be observed in a wide range of biological processes occurring at a multiplicity of spatial scales. A comprehensive review about chemotactic processes can be found in the book by Eisenbach and Lengeler (2004).

Only recently, bacterial chemotaxis in porous media has received attention. Olson (2004) observed for the first time the chemotactic behavior of bacteria in a packed column after applying magnetic resonance imaging. Afterwards, enhancement in bacteria migration due to chemotaxis has been observed in column experiments (Long and Ford 2009; Wang and Ford 2009). For example, Wang and Ford (2009) investigated bacteria migration toward contaminants in porous media under steady flow conditions, while Wang and Ford (2010) quantified transverse (with respect to the mean flow direction) bacteria migration. In another experimental work, Long and Ford (2009) observed, and quantified, the enhancement of transverse dispersion due to the chemotactic behavior of bacteria with respect to a non reactive case. These experimental studies show that bacteria can reach low hydraulic permeability areas, where the contaminants are trapped, thereby including this mechanism is important in modeling studies of bioremediation and natural attenuation of contaminated aquifers.

From the modeling point of view, Phillips et al. (1994) developed a random walk scheme to analyze swimming of bacteria as chemotactic response to local concentration gradients and Kirk and Ginn (2001) studied chemotaxis at the pore scale combining Monte Carlo simulations with cellular dynamics and colloid filtration theories. In successive works, Hilpert (2005) and Long and Hilpert (2008) simulated bacteria chemotaxis in bulk liquids by means of Lattice-Bolzmann to explore contaminant degradation by chemotactic bacteria and the formation of moving bacterial bands, while Valdés-Parada et al. (2009) derived an effective theoretical model to describe chemotaxis coupled to advection and diffusion in porous media at the Darcy’s scale. Finally, Porter et al. (2011) developed a multiscale model of chemotaxis in porous media where transport of bacteria is expressed in terms of effective medium parameters. Both experimental and theoretical works suggest that chemotaxis is an important controlling factor of microbial dynamics in porous media. However, flow fields in porous media display a complex topology and are highly variable in terms of magnitude and direction, leading to the occurrence of nonlinear effects on bacteria motility. This complexity limited so far our understanding and modeling capability of chemotaxis in porous media. In order to properly model transport of microorganisms and their response to the attractants, numerical schemes should accurately reproduce low concentrations and concentration gradients. In particular, the correct computation of concentration gradients which drive chemotaxis represents a challenge for numerical schemes.

A number of numerical models for chemotaxis in porous media are based on Finite Elements (FE) and Finite Volume (FV) schemes, [see for example Blackburn et al. 1997; Bosma et al. 1988; Dillon and Fauci 2000; Nakaguchi and Yagi 2001; Tyson et al. 2000; Zhu and Murray 1995; Ward et al. 2011; Widman et al. 1997]. These well established numerical schemes suffer of artificial numerical diffusion, leading to significant errors in the reproduction of solute gradients, thereby hampering their ability to correctly reproduce the movement of chemotactic bacteria. Innovative numerical schemes, such as Smooth Particle Hydrodynamics (SPH) which minimizes numerical diffusion also for advection dominating flow systems (Boso et al. 2013), are therefore beneficial for studying chemotaxis in porous media at all relevant scales.

SPH is a meshless numerical scheme where the continuum is discretized with a set of particles carrying solute concentrations (Herrera et al. 2010). Contrary to particle tracking, which is based on the assumption that a particle carries a given mass, SPH does not have a minimum detectable concentration, thereby resulting in a better representation of low concentrations (Boso et al. 2013; Herrera et al. 2009, 2010). In addition, diffusion and reactions are computed through mass exchange among neighbouring particles in a fully meshless setup. These features are very appealing for the study of chemotaxis in porous media. However, extending the standard SPH to chemotaxis is not straightforward because bacteria are affected by two velocity fields, advection and chemotaxis velocity, while the chemo-attractant is affected only by advection. Furthermore, SPH does not reproduce correctly concentration gradients, particularly when particles, representing elements of the carrier fluid, are non-uniformly distributed, as it occurs in heterogeneous velocity fields (Boso et al. 2013; Avesani et al. 2014, 2015). Furthermore, an interpolation scheme should be introduced to compute the concentration of the chemoattractant at bacteria positions, which is an additional source of numerical error.

Motivated by these considerations, the primary goal of this paper is to extend a new class of Smooth Particle Hydrodynamics schemes, hereafter referred as MWSPH, developed by Avesani et al. (2014, (2015), to model chemotaxis. MWSPH, in fact, enjoys the following advantages with respect to Eulerian schemes: (i) like traditional streamlines methods, it minimizes artificial numerical diffusion and therefore it is well suited to simulate solute transport in porous media; (ii) it provides a robust mechanism to incorporate chemotaxis within the SPH scheme; (iii) it accurately computes concentration gradients, regardless of the spatial distribution of the particles. The main contribution of this paper is in deriving a new formulation of MWSPH for modeling chemotaxis and demonstrating how it can be used to obtain accurate solutions of the transport equation for chemotactic agents. The numerical scheme is not limited to transport in porous media and can be applied to all cases in which transport of the chemotactic agent is controlled by the advection diffusion equation generalized to include chemotactic behavior. We call this numerical method MWSPH because it combines Moving-Least-Squares (MLS) method of reconstructing continuous functions from known points values with WENO (Weighted Essentially Non-Oscillatory) and SPH schemes (Avesani et al. 2014; Shu 1998).

The paper is organized as follows: Sect. 2 briefly presents the mathematical model of chemotaxis in porous media. Section 3, presents the standard SPH describing its limits in modeling transport with chemotaxis. Section 4 describes how MWSPH incorporates chemotaxis within the SPH framework. Section 5 shows the performance of our new SPH scheme against two analytical solutions and presents a numerical experiment to illustrate its capability to accurately model chemotaxis. Finally, a general discussion and some concluding remarks close the paper in Sect. 6.

## Mathematical modeling

The governing equations of transport in porous media of a passive, i.e. non-reactive, attractant and a chemotactic agent are (Valdés-Parada et al. 2009):

1 $$\begin{aligned} \frac{\partial c_a}{\partial t} + \nabla \cdot \left( \mathbf v _f c_a\right) = \nabla \cdot \left( \mathcal {D}_a \nabla c_a\right) , \end{aligned}$$

for the attractant and

2 $$\begin{aligned} \frac{\partial c_b}{\partial t} + \nabla \cdot \left( \mathbf v _f c_b\right) + \nabla \cdot \left( \mathbf v _c c_b\right) = \nabla \cdot \left( \mathcal {D}_b \nabla c_b\right) , \end{aligned}$$

for the chemotactic agent. In Eqs. (1)–(2) $\mathbf v _f$ is the fluid velocity, $\mathbf v _c$ is the chemotactic velocity, $\mathcal {D}_a$ is the diffusion coefficient of the attractant and $\mathcal {D}_b$ is the diffusion coefficient sum of the passive and active, i.e. chemotactic, diffusions. The concentrations of the attractant and the chemotactic agent are indicated with $c_a$ and $c_b$, respectively. Among the available formulations of $\mathbf v _c$, which represents the motility of the bacteria triggered by the stimulus of the chemo-attractant (e.g. Keller and Segel 1971a, b; Odell and Keller 1976; Chen et al. 1998; Ford and Harvey 2007), we selected the following expression suggested by Chen et al. (1998) and Rivero et al. (1989):

3 $$\begin{aligned} \mathbf v _c = \frac{2}{3} v_s tanh\left( \frac{\chi _0}{2v_s} \frac{k_d \Vert \nabla c_a\Vert }{\left( k_d +c_a\right) ^2}\right) \frac{\nabla c_a}{\Vert \nabla c_a\Vert }; \end{aligned}$$

where $v_s$ is the mean bacteria swimming speed, $\chi _0$ is the chemotactic sensitivity coefficient and $k_d$ is the dissociation constant, representing the ability of the bacteria to sense gradients of the attractant in the surrounding (Segel et al. 1977). In order to apply the SPH formalism Eqs. (1)–(2) are recast in the following Lagrangian form:

4a $$\begin{aligned}&\displaystyle \frac{dc_a}{dt}=\nabla \cdot \left( \mathcal {D}_a \nabla c_a\right) ; \end{aligned}$$

4b $$\begin{aligned}&\displaystyle \frac{d \mathbf r }{d t}=\mathbf v _f; \end{aligned}$$

for the attractant, and

5a $$\begin{aligned}&\displaystyle \frac{dc_b}{dt}=\nabla \cdot \left( \mathcal {D}_b \nabla c_b\right) ; \end{aligned}$$

5b $$\begin{aligned}&\displaystyle \frac{d \mathbf r }{d t}=\mathbf v _f+\mathbf v _c; \end{aligned}$$

for the bacteria. Here $\displaystyle \frac{d}{dt}$ denotes the total time derivative and $\mathbf r$ is the position of the infinitesimal control volume. Equations (4a)–(4b) and (5a)–(5b) represent transport at the pore scale, and should be upscaled to the Darcy’s scale to obtain a model applicable to field-scale simulations. The following Darcy scale equations have been obtained by Valdés-Parada et al. (2009), by applying the volume averaging method to Eqs. (4a)–(4b) and (5a)–(5b):

6a $$\begin{aligned}&\displaystyle \frac{dC_a}{dt}=\nabla \cdot \left( \mathbf D _a \cdot \nabla C_a\right) ; \end{aligned}$$

6b $$\begin{aligned}&\displaystyle \frac{d \mathbf r }{d t}=\mathbf V _f; \end{aligned}$$

for the attractant, and

7a $$\begin{aligned}&\displaystyle \frac{dC_b}{dt}=\nabla \cdot \left( \mathbf D _b \cdot \nabla C_b\right) ; \end{aligned}$$

7b $$\begin{aligned}&\displaystyle \frac{d \mathbf r }{d t}=\mathbf V _f+\mathbf V _c; \end{aligned}$$

for the bacteria, respectively. In Eqs. (6a), (6b), (7a) and (7b) $\mathbf V _f$ is fluid velocity at the Darcy’s scale, while $C_a$ and $C_b$ are the Darcy scale concentrations of attractant and bacteria, respectively. All the other parameters are the upscaled versions, at the Darcy’s scale, of the corresponding quantities defined at the pore scale. $\mathbf V _c$ denotes the chemotactic velocity at Darcy’ scale, while $\mathbf D _a$ and $\mathbf D _b$ are the dispersion tensors for the attractant and the bacteria, both assuming the following form (Bear 1979):

8 $$\begin{aligned} \mathbf {D}_s = (\alpha _{T_s} |\mathbf{V }_s| + \mathcal {D}_{m,s}) \mathbf {I} + (\alpha _{L,s} - \alpha _{T,s}) \frac{\mathbf{V }_s \cdot \mathbf{V }'_s}{|\mathbf{V }_s|}; \end{aligned}$$

with $s=a$ and *b* for the attractant and the bacteria, respectively. In Eq. (8) $\alpha _{L,s}$ and $\alpha _{T,s}$ indicate the longitudinal and transverse dispersivity, respectively. $\mathcal {D}_{m, a}=\mathcal {D}_a$ is the attractant molecular diffusion and $\mathcal {D}_{m, b}=\mathcal {D}_b$ is the random bacteria mobility coefficient. In addition, $\mathbf V _s=\mathbf V _f$ for attractant and $\mathbf V _s=\mathbf V _f+\mathbf V _c$ for bacteria, and $\mathbf V '_s$ denotes the transpose of the vector $\mathbf V _s$. Chemoattractant and bacteria are characterized by different dispersivity tensors and move at a different velocity, as an effect of the autonomous motility of the bacteria.

In most applications, the pore-scale chemotactic velocity (3) is not upscaled at the Darcy’s scale and the scale effect is introduced indirectly by adjusting the parameters through a fitting procedure. For example, Long and Ford (2009) increased arbitrary by two orders of magnitude the sensitive coefficient $\chi _0$ in Eq. (3) in order to reproduce the transverse migration of bacteria observed in their Darcy’s scale experiments. Only recently, Valdés-Parada et al. (2009) and Porter et al. (2011) derived the following effective chemotaxis velocity $\mathbf V _c$ at Darcy’s scale by means of volume averaging of the constitutive equations (Whitaker 1998):

9 $$\begin{aligned} \mathbf{V }_c = \frac{2}{3} v_s tanh\left( \frac{\chi _0}{2 v_s} \frac{k_d \Vert \nabla C_a\Vert }{\left( k_d +C_a\right) ^2}\right) \frac{\nabla C_a}{\Vert \nabla C_a\Vert } \cdot \frac{\mathbf{D }_a}{\mathcal {D}_a}. \end{aligned}$$

The upscaling procedure did not change the nature of the constitutive equations, which are parabolic at both scales, (see the Advection Diffusion Equations (4)–(5) and (6)–(7) written at the pore and Darcy’s scales, respectively) (Evans 2010). Notice that at the Darcy’s scale the effect of small scale velocity fluctuations is represented by an enhanced diffusion tensor resulting from the closure of the pore-scale transport equations (see e.g. Dagan 1989), which is indicated as hydrodynamic dispersion in order to distinguish it from molecular diffusion.

## SPH formulation

The standard SPH formulation of the Advection-Diffusion-Equation (ADE) of the attractant at Darcy’s scale, written in the Lagrangian form of Eqs. (6a) and (6b), is the following (Herrera et al. 2009; Español and Revenga 2003):

10 $$\begin{aligned}&\displaystyle \frac{d C_{a,i}}{dt} = \frac{1}{2} \sum _j^N \frac{m_j}{\rho _{ij}}(C_{a,i}-C_{a,j}) \mathfrak {D}_s(\mathbf r _i,\mathbf r _j) \cdot \nabla _i W_{ij}; \end{aligned}$$

11 $$\begin{aligned}&\displaystyle \frac{d \mathbf r _{i}}{dt}=\mathbf V _{f,i}; \end{aligned}$$

with

12 $$\begin{aligned} \mathfrak {D}_a(\mathbf r _i,\mathbf r _j) = \sum _{l=1}^{\upsilon } \sum _{k=1}^{\upsilon } \left( D_{a,lk}^i + D_{a,lk}^j\right) \left[ 4 \frac{(\mathbf r _j-\mathbf r _i)_{l}(\mathbf r _j-\mathbf r _i)_{k}}{|\mathbf r _j-\mathbf r _i|^2} \right] -\delta _{lk}, \end{aligned}$$

where $C_{a,i}$ and $C_{a,j}$ are the attractant concentrations of the particles *i* and *j* located at $\mathbf r _i$ and $\mathbf r _j$, respectively, $m_i$ is the mass of the $i-$th particle, $\rho _i$ is its density, $\upsilon$ is the space dimensionality, in this case $\upsilon =2$, $\mathbf V _{f,i}$ is the fluid velocity of the $i-th$ particle and $\rho _{ij}=(\rho _i+\rho _j)/2$. In addition, $D_{a,lk}$ indicates the component *l*, *k* of the dispersion tensor $\mathbf {D}_{a}$ and $\delta _{ij}$ is the Kronecker’s delta (Avesani et al. 2015). The density $\rho _i$ evolves in time depending on the relative positions of the particles:

13 $$\begin{aligned} \rho _i=\sum _{j=1}^N m_j W_{ij}. \end{aligned}$$

The term $W_{ij}$ is the interpolating kernel function centered in $\mathbf r _i$ with respect to the location $\mathbf r _j$. As suggested by Ferrari et al. (2009), we use the cubic B-spline, which is defined as follows:

14 $$\begin{aligned} W_{ij} =\frac{\mathcal {C}}{h_{ij}^\upsilon } {\left\{ \begin{array}{ll} 1/3 -q_{ij}^2+q_{ij}^3/2 &{} \text {if } 0 \le q_{ij} < 1 ; \\ (2 -q_{ij})^2+q_{ij}^3 &{} \text {if } 1 \le q_{ij} \le 2; \\ 0 &{} \text {if } q_{ij} > 2 ; \end{array}\right. } \end{aligned}$$

where $q_{ij}=|r_j-r_i|/h_{ij}$ and $\mathcal {C}$ is the normalization constant so that $\int W_{ij}=1$. The term $h_{ij}$ is the smoothing length, which is locally variable:

15 $$\begin{aligned} h_{ij}=\frac{1}{2}\left( h_i +h_j\right) ,\qquad \text {with}\qquad h_i= \sigma \root \upsilon \of {\frac{m_j}{\rho _j}}. \end{aligned}$$

Similarly, in the SPH formalism, the Eqs. (7a) and (7b) for bacteria including chemotaxis read as follows:

16 $$\begin{aligned} \frac{d C_{b,i}}{dt} = \frac{1}{2} \sum _j^N \frac{m_j}{\rho _{ij}}(C_{b,i}-C_{b,j}) \mathfrak {D}_b(\mathbf r _i,\mathbf r _j) \cdot \nabla _i W_{ij}, \end{aligned}$$

and

17 $$\begin{aligned} \frac{d \mathbf r _{i}}{dt}=\mathbf V _{f,i}+\mathbf V _{c,i}, \end{aligned}$$

where

18 $$\begin{aligned} \mathfrak {D}_b(\mathbf r _i,\mathbf r _j) = \sum _{l=1}^{\upsilon } \sum _{k=1}^{\upsilon } \left( D_{b,lk}^i + D_{b,lk}^j\right) \left[ 4 \frac{(\mathbf r _j-\mathbf r _i)_{l}(\mathbf r _j-\mathbf r _i)_{k}}{|\mathbf r _j-\mathbf r _i|^2} \right] -\delta _{lk}. \end{aligned}$$

In this case, $D_{b,lk}$ are the component *l*, *k* of the bacterial dispersion tensor $\mathbf {D}_{b}$ and $\mathbf V _{c,i}$ is chemotactic velocity of the $i-th$ particle.

In the standard SPH formulation, the particles represent volumes of fluid with concentration that varies in time as the particles move according to the flow field. While the attractant is influenced by the fluid velocity field, the bacteria feel the superposition of the fluid and chemotactic velocities, the latter is controlled by the attractant concentration gradient. To address transport of both bacteria and attractant with the classical SPH scheme, two sets of particles should be used, which occupy different positions within the computational domain because tracked with different velocity fields. As a consequence, an interpolation scheme should be devised in order to transfer the information on attractant concentration from the attractant particles to the positions occupied by the bacteria particles. This operation inevitably introduces smoothing in the attractant concentration distribution, thereby introducing unwanted errors in concentration gradient, and consequently in the computed chemotactic velocity. In addition, the bacteria particles tend to cluster where the concentration of the attractant is larger (moving towards the attractant concentration gradient), leading to an uneven distribution of the bacteria, which has been shown to be a source of large errors when transport is modeled by using the standard SPH (Boso et al. 2013; Avesani et al. 2015; Herrera et al. 2009). Furthermore, clustering of bacteria particles generates zones in the domain with a low particle density; when the distance between the particles is of the same order, or larger, than the smoothing length, concentration is poorly estimated, such as its gradient (Herrera et al. 2009).

Consequently, extension of standard SPH to model both attractant and bacterial concentrations is not straightforward and error prone. Furthermore, a set of particles should be used for each bacteria species that is modeled.

## The new SPH scheme

In this section, we extend the MWSPH scheme, developed by Avesani et al. (2014, (2015), to include chemotaxis. MWSPH, which is an evolution of the method developed by Vila (1999) and Ben Moussa et al. (2006), uses a high order Riemann solver to evaluate numerical fluxes between interacting particles (Avesani et al. 2014). We take advantage of the fact that in SPH the particle represents a small volume of fluid to envision a new meshless scheme. The key idea is to develop a meshless method in which a single set of particles carry the information of both attractant and bacterial concentrations.

**Fig. 1:**
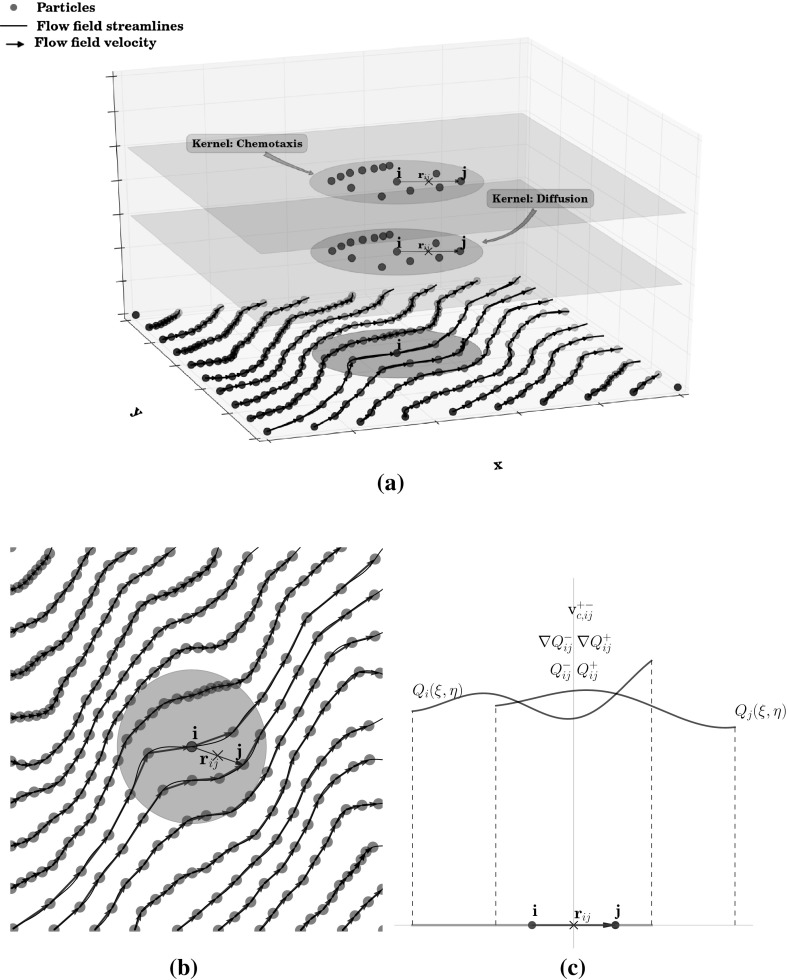
Meshless MWSPH scheme extended to chemotaxis. Particles move along streamlines of the flow field carrying both bacterial and attractant concentrations. Each particle exchanges mass with other particles contained into its kernel support to model both for dispersion and chemotaxis (advective) fluxes. **c** A one-dimensional section through the reconstruction polynomials along the *line* connecting particles $\mathcal {P}_i$ and $\mathcal {P}_j$ as well states $Q_{ij}^-$,$\nabla Q_{ij}^-$and $Q_{ij}^+$,$\nabla Q_{ij}^+$extrapolated to the midpoint and chemotactic velocity computed form the extrapolated states

Figure 1 shows a sketch explaining this new scheme: particles move according to the flow field carrying both bacterial and attractant concentrations while exchanging mass due to diffusion and chemotaxis. These fluxes are estimated by using a high-order polynomial reconstruction of concentrations in a full meshfree scheme. The diffusive and chemotactic fluxes are evaluated at the midpoint between two interacting particles using a Riemann solver. Notice that, once the concentration of the attractant and its gradient have been estimated at the mid point of the segment connecting the two particles, the chemotactic velocity can be computed at the same position by means of Eq. (9). The high order reconstruction polynomials together with the Riemann solver allow to capture the sharp bacterial concentration front caused by chemotactic velocity.

According to this new scheme, the general advection-diffusion equation at Darcy’s scale, including chemotaxis, can be rewritten as follows:

19 $$\begin{aligned} \frac{d}{dt}\mathbf{r }=\mathbf{V }_{f}, \end{aligned}$$

and

20 $$\begin{aligned} \frac{d}{dt}\mathbf{Q } = \nabla \left( \mathbf{F }(\mathbf{Q },\nabla \mathbf{Q }) \right) , \end{aligned}$$

where $\mathbf Q =(C_a,C_b)$ is the vector of the attractant and bacterial concentrations and $\mathbf F (\mathbf Q ,\nabla \mathbf Q )$ is the nonlinear flux vector, which is given by:

21 $$\begin{aligned} \mathbf F (\mathbf Q ,\nabla \mathbf Q )= \left[ \begin{array}{c} -\mathbf D _a \cdot \nabla C_a\\ -\mathbf D _b \cdot \nabla C_b + \mathbf V _c C_b \end{array} \right] . \end{aligned}$$

The new MWSPH scheme, including chemotaxis, assumes then the following form:

22 $$\begin{aligned}&\displaystyle \frac{d \left( V \mathbf Q \right) _i}{dt}=-\sum _{j=1}^N V_i V_j 2\mathbf G _{ij} \cdot \nabla W_{ij}, \end{aligned}$$

23 $$\begin{aligned}&\displaystyle \frac{d V_i}{dt}=\sum _{j=1}^N \left( \bar{\mathbf{V }}_{ij}-\mathbf V _{f,i}\right) \cdot \nabla W_{ij}, \end{aligned}$$

24 $$\begin{aligned}&\displaystyle \frac{d \mathbf r _i}{dt}=\mathbf V _{f,i}, \end{aligned}$$

where $\mathbf G _{ij}$ is the numerical flux tensor, $V_i$ is the particle volume, and $\bar{\mathbf{v }}_{ij}$ is the velocity at the interface between the two interacting particles $\mathcal {P}_i$ and $\mathcal {P}_j$. Equation (23) takes into account the deformation of the fluid element caused by the spatially non-uniform velocity field (Ferrari et al. 2009). Following Avesani et al. (2014), the numerical flux $\mathbf G _{ij}$ is approximated by the Rusanov-type flux (Dumbser et al. 2008; Dumbser 2010) as

25 $$\begin{aligned} \mathbf G _{ij}=\frac{1}{2} \left( \mathbf H _i\left( \mathbf Q _{ij}^-,\nabla \mathbf Q _{ij}^-,\bar{\mathbf{V }}_{f,ij}\right) + \mathbf H _j\left( \mathbf Q _{ij}^+,\nabla \mathbf Q _{ij}^+, \bar{\mathbf{V }}_{f,ij}\right) \right) - \varTheta \left( \mathbf Q _{ij}^+ - \mathbf Q _{ij}^- \right) \otimes \mathbf n _{ij}, \end{aligned}$$

with

26 $$\begin{aligned} \bar{\mathbf{V }}_{f,ij}=\frac{1}{2}\left( \mathbf V _{i}^-+ \mathbf V _{j}^+\right) ; \end{aligned}$$

where $\mathbf n _{ij}$ the unitary vector of the distance between particles $\mathcal {P}_i$ and $\mathcal {P}_j$ and $\mathbf H$ the flux tensor in the reference frame moving with velocity $\mathbf V _f$:

27 $$\begin{aligned} \mathbf H \left( \mathbf Q ,\nabla \mathbf Q ,\mathbf v _f \right) = \mathbf F \left( \mathbf Q ,\nabla \mathbf Q \right) -\mathbf Q \otimes \mathbf V _f; \end{aligned}$$

where the chemotactic velocity is computed from the extrapolated left and right states, indicated with the superscript “−” and “$+$”, respectively. In Eq. (25) the term $\varTheta$ represents the dissipation matrix (Hidalgo and Dumbser 2011):

28 $$\begin{aligned} \varTheta =\left( \lambda _{ij} - \frac{1}{\Vert \mathbf r \Vert } \lambda _{v_s,ij}\right) \mathbf I \end{aligned}$$

where $\lambda _{ij}$ is the maximum absolute eigenvalue of the Jacobian matrix of the convective flux evaluated with respect to $\mathbf Q$, while $\lambda _{v_s,ij}$ is the maximum absolute eigenvalue of the Jacobian matrix of the diffusive flux evaluated with respect to $\nabla \mathbf Q$. $\lambda _{ij}$ and $\lambda _{v_s,ij}$ are evaluated along $\mathbf n _{ij}$ in a moving frame. In the first case $\lambda _{ij}$ reads as:

29 $$\begin{aligned} \lambda _{ij}=\max (|\varvec{\varLambda }_i^-|,|\varvec{\varLambda }_j^+|), \end{aligned}$$

with $\varvec{\varLambda }$ being the diagonal matrix of eigenvalues of $\mathbf A _\mathbf n (\mathbf Q ,\mathbf v )=\partial \mathbf H / \partial \mathbf Q \cdot \mathbf n _{ij}$. In the second $\lambda _{v_s,ij}$ is defined as:

30 $$\begin{aligned} \lambda _{v_s,ij}=\max (|\varvec{\varLambda }_{v_s,i}^-|,|\varvec{\varLambda }_{v_s,j}^+|), \end{aligned}$$

with $\varvec{\varLambda _{v_s}}$ being the diagonal matrix of eigenvalues of $\mathbf B _\mathbf n (\nabla \mathbf Q ,\mathbf v )=\partial \mathbf H / \partial (\nabla \mathbf Q ) \cdot \mathbf n _{ij}$. Furthermore, $\mathbf Q _{ij}^-$ and $\mathbf Q _{ij}^+$ are the left and right states at the midpoint of the two interacting particles obtained by the high order MLS-WENO reconstruction polynomials $\mathbf Q _{i}(\mathbf r )$ for particle $\mathcal {P}_i$ and $\mathbf Q _{j}(\mathbf r )$ for particle $\mathcal {P}_j$, both of order *M*. Additional details on the MLS-WENO reconstruction scheme are reported in the work by Avesani et al. (2014).

For sake of clarity, in the present work we have limited ourself to one attractant and one bacteria species. However, the effort to include more bacteria species is limited because all the information is contained in the same set of particles. This is an important advantage with respect to the standard SPH, which, as explained before, requires a set of particles for each bacterial species.

## Test cases

In this section, we evaluate the accuracy of the proposed new numerical scheme by using a few test cases. In all test cases time step has been computed as follows:

31 $$\begin{aligned} dt=CFL \frac{h_{ij}^{min}}{|\lambda _{ij}^{max}|+2 |\lambda _{v_s,ij}^{max}|/h_{ij}^{min}}, \end{aligned}$$

where CFL is the Courant number according to the Courant-Friedrichs-Lewy condition (Courant et al. 1928). Following Ferrari et al. (2009) and Avesani et al. (2014), Eqs. (22) and (23) are solved by using an explicit third order Runge-Kutta scheme in time, which ensures linear stability of the numerical solution, while Eq. (24) is solved by using a standard particle tracking scheme.

### One dimensional test case

Let us consider transport at the pore scale of a chemoattractant and the associated bacteria population in a one-dimensional domain and in the absence of fluid advection, as described by the following set of equations:

32 $$\begin{aligned} \frac{\partial c_a}{\partial t}=\mathcal {D}_a\frac{\partial ^2 c_a}{\partial x^2}+r_s; \end{aligned}$$

for the chemoattractant, and

33 $$\begin{aligned} \frac{\partial c_b}{\partial t}+ \frac{\partial }{\partial x} \left( v_c c_b\right) =\mathcal {D}_b \frac{\partial ^2 c_b}{\partial x^2}-Yr_s; \end{aligned}$$

for the bacteria. In the Eq. (33), *Y* is the bacterial growth factor and $r_s$ the reaction rate regulating the consumption of the chemical attractant, which is given by the following Monod type expression (Monod 1949):

34 $$\begin{aligned} r_s=-q\frac{c_a}{c_a+k_s}c_b, \end{aligned}$$

where $k_s$ is the half saturation constant and *q* is the maximum reaction rate between the bacteria and the attractant. The chemotactic velocity is related linearly to the concentration gradient of the attractant through the following expression:

35 $$\begin{aligned} v_c=\frac{\chi _0 k_d }{3 (k_d + c_a)^2} \frac{\partial c_a}{\partial x}. \end{aligned}$$

We selected this equation to parametrize chemotactic velocity because it allows to solve analytically the Eqs. (32) and (33) and exerts a similar effect of Eq. (3) on bacteria motility. An in depth discussion of this model is given by Alt (1980), Erban and Othmer (2004) and Ford and Harvey (2007).

Long and Hilpert (2007) showed that the model described by the Eqs. (32) and (33), with the following boundary conditions:

36a $$\begin{aligned}&\displaystyle \frac{\partial c_a}{\partial x}= {\left\{ \begin{array}{ll} 0 &{} \text {for } x = -\infty ; \\ 0 &{} \text {for } x = +\infty ; \end{array}\right. } \end{aligned}$$

36b $$\begin{aligned}&\displaystyle c_a= {\left\{ \begin{array}{ll} 0 &{} \text {for } x = -\infty ; \\ c_{a0} &{} \text {for } x = +\infty ; \end{array}\right. } \end{aligned}$$

37a $$\begin{aligned}&\displaystyle \frac{\partial c_b}{\partial x}= {\left\{ \begin{array}{ll} 0 &{} \text {for } x = -\infty ; \\ 0 &{} \text {for } x = +\infty ; \end{array}\right. } \end{aligned}$$

37b $$\begin{aligned}&\displaystyle c_b= {\left\{ \begin{array}{ll} c_{b0} &{} \text {for } x = -\infty ; \\ 0 &{} \text {for } x = +\infty . \end{array}\right. } \end{aligned}$$

Under the additional assumptions that the bacteria population remains stable (i.e. $Y=0$), while the attractant is consumed at the rate $r_s$, given by Eq. (34) by bacteria moving with the chemotactic velocity (35), Eqs. (32) and (33) admit the following traveling wave analytical solutions for the concentration of the bacteria

38 $$\begin{aligned} \frac{c_b}{c_{b0}}=\left( \frac{c_a}{c_{a0}}\right) ^{\varUpsilon } \exp \left( -\frac{ c}{\mathcal {D}_b} \left( x-ct\right) \right) ; \end{aligned}$$

and the attractant

39 $$\begin{aligned} \mathcal {F}\left( \frac{c_a}{c_{a0}}\right) - \mathcal {F}\left( 1 \right) = \frac{c_{b0} q \mathcal {D}_b}{ c^2 c_{a0}} \exp \left( -\frac{c}{\mathcal {D}_b} \left( x-ct\right) \right) ; \end{aligned}$$

where *c* is the celerity of propagation of the traveling waves (see below), $\varUpsilon =\varLambda /\mathcal {D}_b$ is a constant of integration where $\varLambda$ is a constant with dimensions $[L^2/T]$. In this one dimensional test case we set $\varUpsilon =6$, according to Long and Hilpert (2007). Finally, $\mathcal {F}(\mathcal {\chi })$ is a function defined as follows

40 $$\begin{aligned} \mathcal {F}(\mathcal {\chi }) = \frac{\mathcal {\chi }^{1-\varUpsilon }}{\varUpsilon -1} + \frac{k_s}{c_{a0}} \frac{\mathcal {\chi }^{-\varUpsilon }}{\varUpsilon }. \end{aligned}$$

The Eqs. (38) and (39) describe two concentration bands traveling at a constant speed *c*, given by:

41 $$\begin{aligned} c= \sqrt{\frac{c_{b_0}q \mathcal {D}_b}{ \varUpsilon }} \end{aligned}$$

The terms $c_{a_0}$ and $c_{b_0}$ are the maximum initial concentrations for attractant and bacteria, respectively. The parameters used in the numerical experiment are reported in the Table 1 and are reproduced form Pedit et al. (2002). The reactive term $r_s$ is solved with an implicit Newton-Raphson scheme (Press et al. 1992).

**Table 1:** The parameters used in one dimensional Long and Hilpert test case

Parameter	Symbol	Value	Unit
Initial bacterial concentration	$c_{b0}$	$4.9\text{ e }+9$	cfu/l
Initial attractant concentration	$c_{a0}$	$2.83\text{ e }-6$	g/l
Attractant diffusion coefficient	$\mathcal {D}_a$	$7.5\text{ e }-10$	m$^2$/s
Bacteria diffusion coefficient	$\mathcal {D}_b$	$3.2\text{ e }-11$	m$^2$/s
Maximum rate of attractant consumption	*q*	$7.9e\text{ e }-16$	g/cfu/s
Yield coefficient	*Y*	0	g/cfu/s
Dissociation constant	$k_d$	$2.1\text{ e }-3$	g/l
Half saturation constant	$k_s$	$1.3\text{ e }-4$	g/l
Chemotactic sensitivity coefficient	$\chi _0$	$1.8\text{ e }-9$	m$^2$/s
Bacteria mean swimming velocity	$v_s$	$48\text{ e }-6$	m/s

**Fig. 2:**
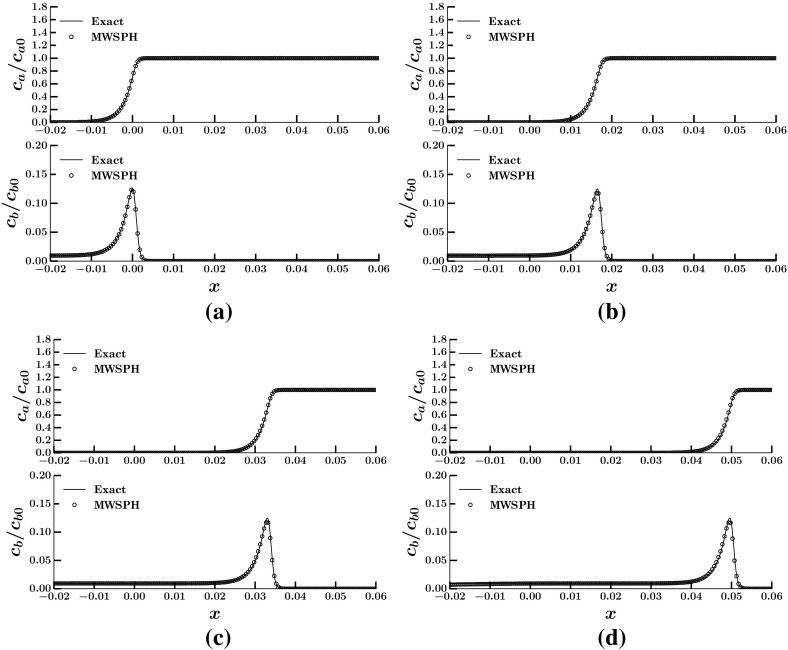
Numerical solution for one dimensional Long and Hilpert test case at a few time step for $CLF=0.8$ $\sigma =2$ and $M=2$. **a** $T=0$. **b** $T=2.88 \cdot 10^{-5}$. **c** $T=6.72 \cdot 10^{-5}$. **d** $T=9.60 \cdot 10^{-5}$

According to Long and Hilpert (2007), Eqs. (38)–(39) represent bacterial and attractant concentration waves that travel without deformation with a constant speed.

Figure 2a–d show both bacterial and attractant concentrations at four times since the injection of bacteria, computed using 400 particles. The numerical solutions obtained with MWSPH reproduce accurately the analytical solutions (38) and (39) for both bacterial and attractant concentrations, at all the explored times. Notice how MWSPH is able to accurately reproduce the sharp front of bacterial concentration. At the dimensionless time $T=9.60 \cdot 10^{-5}$, the errors of the numerical solutions $E=\sqrt{\sum _{j=1}^N e_j^2}/N$, where $e_j$ is the difference between the numerical and the analytical solutions at the $j-$th particle location, are $6.92\,\cdot \,10^{-6}$ for bacteria and $4.64\,\cdot \,10^{-5}$ for attractant. The dimensionless time *T* is defined as $T=t\, v_s/l_0$, where $l_0$ is a suitable characteristic length. Our numerical solutions preserve the initial shape of the traveling waves of both attractant and bacterial concentrations at all the explored times, as required by the analytical solutions and reproduce accurately the maximum concentrations. In particular, the difference between the maximum bacterial concentration computed with MWSPH and obtained from the analytical solution varies between 0.22 and $0.02\,\%$ at the four times shown in Fig. 2a–d.

### Interplay between chemotaxis and diffusion

In this test case we consider an instantaneous release of bacteria and attractant in a homogeneous flow field at Darcy’s scale with velocity $v_0$ tilted of $30^o$ with respect to the x-axis, where the initial attractant and bacterial concentrations are given by:

42 $$\begin{aligned} C_i(x,y)=\frac{m_i}{2\, \pi \, w^2} \exp {\left( \frac{-(x-x_{i,0})^2-(y-y_{i,0})^2}{2\, w^2}\right) }; \end{aligned}$$

where $m_i$ is the released mass per unit of thickness of attractant ($i=a$) and bacteria ($i=b$). The initial maximum concentration of attractant and bacteria occur at different positions $(x,y)=(x_{i,0}, y_{i,0})$, $i=a,b$ and assume the following values: $C_{a,0}=m_a/(2 \pi w^2)$ and $C_{b,0} = m_b/(2 \pi w^2)$ for attractant and bacteria, respectively. The evolution in time of the concentration of both attractant and bacteria are obtained by applying MWSPH to the constitutive Eqs. (4a)–(4b) and (5a)–(5b), as discussed in Sect. 4, and with the parameters reported in Table 2. The dispersion tensors for attractant and bacteria are obtained by substituting the dispersivities shown in Table 2 into the expression (8). Transverse dispersivity $\alpha _{s,T}$ is set to 0.004 cm for attractant (i.e., $s=a$) and 0.0008 cm for bacteria (i.e., $s=b$), according to Long and Ford (2009)’s experiment, and flow velocity, $v_0$, is of the same order of magnitude of that used in laboratory experiments [see for examples Wang and Ford 2009, 2010].

**Fig. 3:**
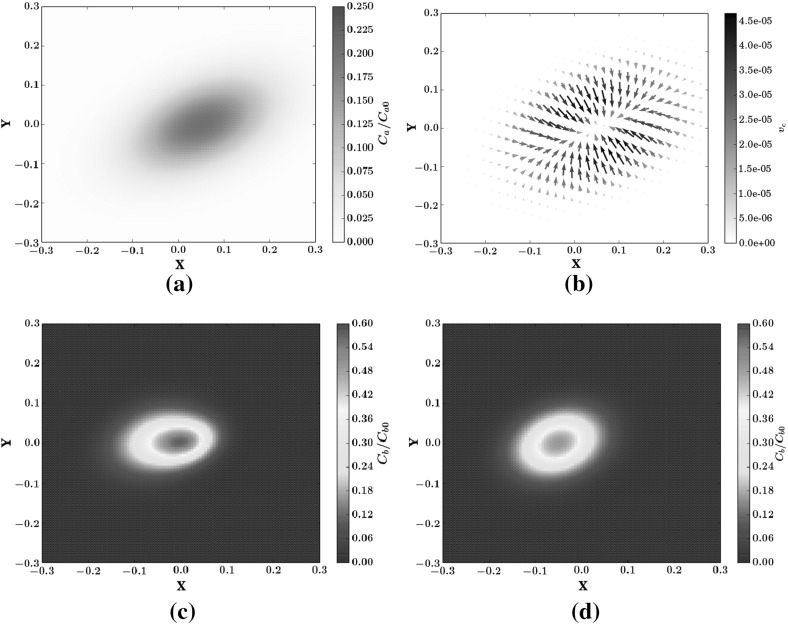
Numeral results of the diffusion test case at dimensionless time $T=3.76$ and for $CFL=0.9$, $\sigma =3$, $\sigma _{mls}=4$. **a** The attractant concentration, **b** the chemotactic velocity field, **c**, **d** the bacterial concentration with and without chemotaxis, respectively. **a** Attractant. **b** Chemotaxis velocity field. **c** Bacteria with chemotaxis. **d** Bacteria without chemotaxis

The purpose of this numerical test is twofold: on one hand we test the numerical scheme in a two-dimensional setup with an anisotropic dispersion tensor, on the other, we study the effects of chemotaxis on bacterial dispersion. From the numerical point of view, an anisotropic dispersion tensor [see Eq. (8)], may lead to spurious oscillations. This is a known problem affecting the standard SPH when applied to the Advection-Diffusion-Equation for a non reactive solute (Herrera and Beckie 2012), which is significantly alleviated by the MWSPH scheme (Avesani et al. 2015). In this test case we investigate whether MWSPH is able to reproduce accurately the distribution of bacterial concentration, which is sensitive to the accuracy with which the attractant concentration gradient is computed. Spurious, though of small amplitude, oscillations of the attractant concentration may lead to large oscillations of the chemotactic velocity with a negative feedback on the distribution of bacterial concentration. Figure 3 illustrates the results of the simulations at the dimensionless time $T= t v_0/w = 3.76$. The numerical solution is free of spurious oscillations, as Fig. 3a, c, d clearly show. Furthermore, the chemotactic velocity field (Fig. 3b) is congruent with the distribution of the attractant shown in Fig. 3a. Figure 3c shows the bacterial concentration at the final simulation time in case the chemotaxis is not taken into account (i.e., for $V_c=0$), while the contribution of the chemotaxis to bacterial transport is shown in Fig. 3c, d. Including chemotaxis leads to a maximum bacterial concentration which is 16 $\%$ higher than in the absence of chemotaxis. Furthermore, the numerical solution captures well the translation of the center of mass of the bacteria towards the center of mass of the attractant, which does not occur in case of non-chemotactic bacteria. Indeed, bacteria moves with a different chemotatic speed according to the attractant gradient and attractant concentration, which values change in space, as shown Fig. 3b, a. Non-uniformity of the chemotactic velocity leads to bacteria plume deformation and a different trajectory of the bacteria center of mass with respect to attractant center of mass.

**Table 2:** The parameters used in the two-dimensional test case

	Symbol	Value	Unit
Attractant parameters			
Attractant maximum initial concentration	$C_{a0}$	$0.3\text{ e- }4$	mg/l
Control of the mass release	*w*	0.04	cm
Attractant initial plume maximum position	$[x_{a,0},y_{a,0}]$	[0.05, 0]	cm
Attractant longitudinal dispersivity	$\alpha _{L,a}$	0.04	cm
Attractant transversal dispersivity	$\alpha _{T,a}$	0.004	cm
Bacteria parameters			
Bacteria maximum initial concentration	$C_b/C_{b0}$	1	
Control of the mass release	*w*	0.04	cm
Bacteria initial plume maximum position	$[x_{b,0},y_{b,0}]$	$[-0.05,0]$	cm
Bacteria longitudinal dispersivity	$\alpha _{L,b}$	0.008	cm
Bacteria transversal dispersivity	$\alpha _{T,b}$	0.0008	cm
Flow field parameters			
Flow field velocity	$v_0$	$1\text{ e- }3$	cm/s
Flow field orientation	$\beta$	30$^{\circ }$	

**Fig. 4:**
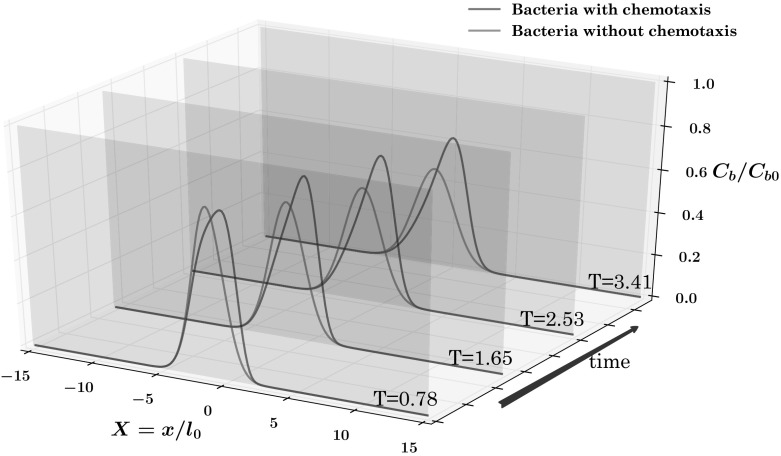
Bacteria concentration and snapshots at different time steps at section $sec-x$ both with and without chemotaxis for the two-dimensional diffusion test case. In all cases $CFL=0.9$, $\sigma =3$ and $\sigma _{mls}=4$

**Table 3:** The parameters for heterogeneous test case

	Symbol	Value	Unit
Attractant parameters			
Attractant maximum initial concentration	$C_{a0}$	$0.3\text{ e- }4$	mg/l
Control of attractant mass release	$l_w$	0.04	cm
Attractant longitudinal dispersivity	$\alpha _{L,a}$	0.04	cm
Attractant transversal dispersivity	$\alpha _{T,a}$	0.004	cm
Initial diffusion time for the attractant	$t^i$	80	s
Bacteria parameters			
Bacteria maximum initial concentration	$C_b/C_{b0}$	1	
Bacteria longitudinal dispersivity	$\alpha _{L,b}$	0.008	cm
Bacteria transversal dispersivity	$\alpha _{T,b}$	0.0008	cm
Chemotactic response parameter			
Bacteria mean swimming velocity	$v_s$	$4.8\text{ e- }3$	cm/s
Chemotatic receptor constant	$k_d$	$1.25\text{ e- }4$	mg/l
Chemotatic sensitivity	$\chi _0$	$8.0\text{ e- }4$	cm$^2$/s
Flow field			
Internal velocity	$V_1$	0.00202	cm/s
External velocity	$V_2$	0.0087	cm/s

These characteristics of the bacteria plume are better illustrated in Fig. 4, which shows bacterial concentration along a line parallel to the *x* axis and passing through the center of the plume at several time-steps. The difference in the bacterial concentration peak when chemotaxis is turned on is evident and it increases with time as the chemotactic effect strengths (Fig. 4).

### Effect of chemotaxis in heterogeneous porous media

This numerical experiment is intended to illustrate the interaction between the chemotactic flow field and the advection flow field at Darcy’s scale when they are solved simultaneously with MWSPH. To simplify the analysis, consumption of the attractant by bacteria is not considered in this example. Table 3 shows the numerical setup adopted in the numerical simulations, which complies with the experimental configuration adopted by Wang and Ford (2009). The set up is composed by a circular “rod” surrounded by a matrix with different hydraulic conductivity. Owing to the radial symmetry of the column, we consider a rectangular domain 20 cm long and 5  cm wide composed by three layers; the hydraulic conductivity of the inner layer is 4.3 times lower than the hydraulic conductivity of the other two layers. Differently from Wang and Ford (2009) we assigned to the inner rod a smaller hydraulic conductivity with respect to the surrounding matrix, such as to mimic the situation typically encountered locally in contaminated aquifers where the attractant is entrapped in low conductivity zones. Consequently, the solutions of our exercise cannot be directly compared with the experimental results of Wang and Ford (2009). A uniform head gradient is applied along the layers in the direction *x* leading to flow velocities of 0.00202 cm/s and 0.0087 cm/s in the inner and outer layers, respectively, in proportion to the imposed hydraulic conductivity (see Table 3).

The initial attractant concentration is given by:

43 $$\begin{aligned} C_a/C_{a0}= {\left\{ \begin{array}{ll} \frac{1}{2}\left( erfc \left( \frac{(y-y_0)-l_w}{\sqrt{4 \mathcal {D}_a t^i}}\right) -erfc \left( \frac{(y-y_0)+l_w}{\sqrt{4 \mathcal {D}_a t^i}}\right) \right) &{} \text {if } 1.5 \le x < 10; \\ 0 &{} \text {otherwise}; \end{array}\right. } \end{aligned}$$

which represents the distribution of attractant concentration resulting from an instantaneous injection at $t=0$ of a solute within a strip of width $l_w$ with longitudinal axis at $y=y_0$, evaluated at $t=t^i$ and sampled between $x=1.5$ and $x=10$. Furthermore, the initial bacterial concentration is set to:

44 $$\begin{aligned} C_b/C_{b0}= {\left\{ \begin{array}{ll} 1 &{} \text {if } 2 \le x < 3; \\ 0 &{} \text {otherwise}. \end{array}\right. } \end{aligned}$$

**Fig. 5:**
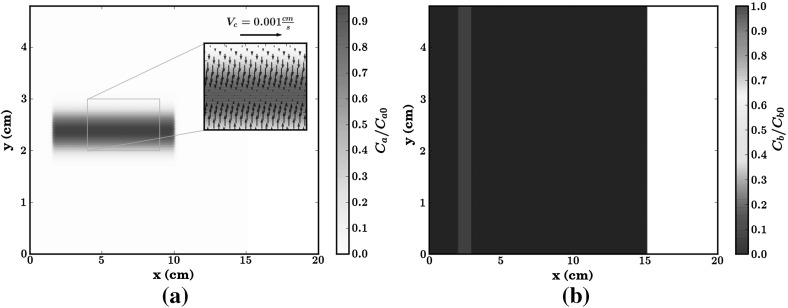
Sketch of the numerical setup for the dual-layer test case, using 30000 particles for $CFL=0.9$, $\sigma =3$ and $\sigma _{mls}=4$. **a** Initial condition attractant and chemotatic velocity field. **b** Initial condition bacteria

The fluid is discretized by using 30, 000 particles uniformly distributed within the domain, each one carrying the information on the concentration of both attractant and bacteria. The parameters controlling transport of the two species are shown in Table 3 and Figure 5 shows the initial numerical experiment set-up.

**Fig. 6:**
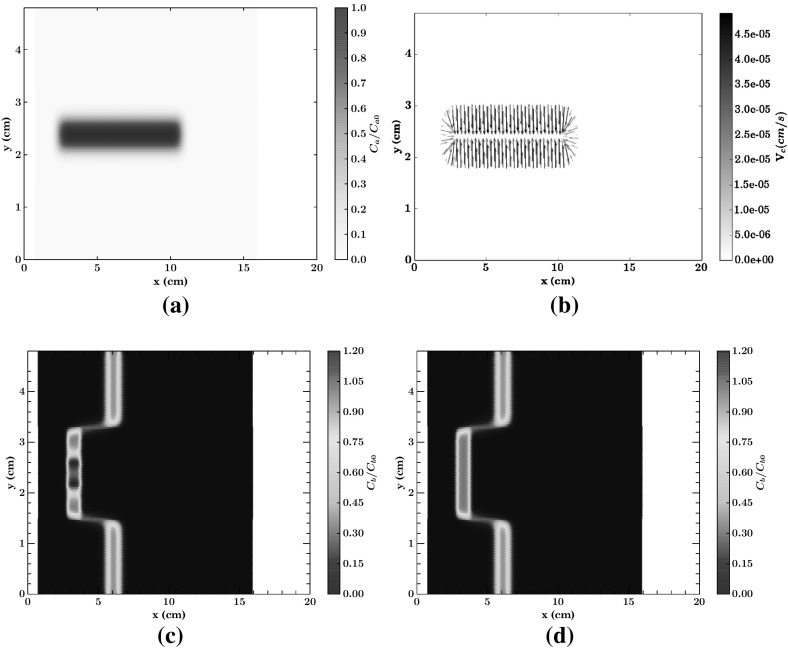
Numerical results for dual-layer test case, $CFL=9$, $\sigma =3$, $\sigma _{mls}=4$. **a** Attractant concentration. **b** Chemotactic velocity field. **c** Bacteria with chemotaxis. **d** Bacteria without chemotaxis

Figure 6a–d show the results of the simulations after $1225\, s$ since the release of bacteria, corresponding to 150 computational time steps. Figure 6c, d underline the difference between bacterial transport with or without the contribution of a chemotactic velocity. Chemotaxis leads to the development of two peaks, which cannot develop in its absence, as shown in Fig. 6c, d. At the final simulation time ($1225\, s$ since the injection of bacteria) the maximum concentration of bacteria is 20 $\%$ higher in the presence of chemotaxis. Correspondingly, the mass of bacteria in the lower permeability layer increases by 12 $\%$. As for the test case presented in Sect. 3, the computed concentration of both bacteria and attractant are free of spurious oscillations, as can be seen in Fig. 6a, c, d.

**Fig. 7:**
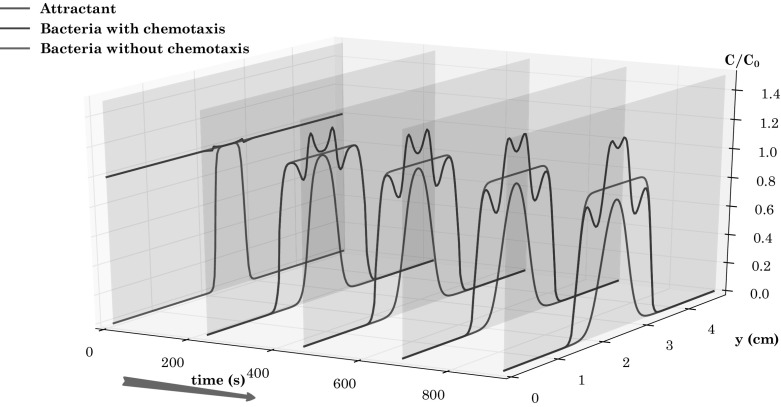
Numerical results for the heterogeneous three-layer test case, $CFL=0.9$, $\sigma =3$, $\sigma _{mls}=4$

In order to better appreciate the movement of bacteria, due to the contribution of chemotaxis, Fig. 7 shows the concentration of bacteria and attractant, the former both in the presence and in the absence of chemotaxis, at different times *t* along the cross section $x=V_1\, t$. The appearance of four peaks in the bacterial concentration is clearly seen, as well as the smooth behavior in the absence of chemotaxis. The concentration values of the two main peaks increase in time and move toward center of the inner layer. These are the so called traveling bacterial bands which have been already observed in a number of experimental works (Keller and Segel 1971a; Rivero et al. 1989; Wang and Ford 2009).

## Conclusion

We presented an alternative formulation of SPH for the numerical solution of transport of chemotactic bacteria in porous media based on the MWSPH scheme developed by Avesani et al. (2014, (2015). This new numerical methods overcomes the limitations of standard SPH, which cannot efficiently (and accurately) handle multiple advective fields like in the case of chemotaxis. Our new numerical method, uses a single set of particles and permits to handle any number of bacteria species and attractants. Furthermore, it is accurate in the reproduction of both the concentration and the concentration gradient of the attractant, thereby leading to an accurate reproduction of bacteria motility. The sharp variation of bacterial concentration is adequately reproduced such as the traveling bands that emerge under particular conditions during chemotactic transport. Furthermore, our method is stable under Courant-Friedrichs-Lewy conditions and do not show spurious oscillations in the numerical solutions. In the present study, we limited ourself to one- and two-dimensional simulations, but the method is general and can be applied also to three dimensional problems and to advection-diffusion-chemotaxis transport with reaction terms.
